# Supplementary figures and images for: Elimination of PCR duplicates in RNA-seq and small RNA-seq using unique molecular identifiers
Source: BMC Genomics. 2018 Jul 13;19:531. doi: 10.1186/s12864-018-4933-1 (PMC6044086; doi:10.1186/s12864-018-4933-1)

No error correction, Error correction, Truth

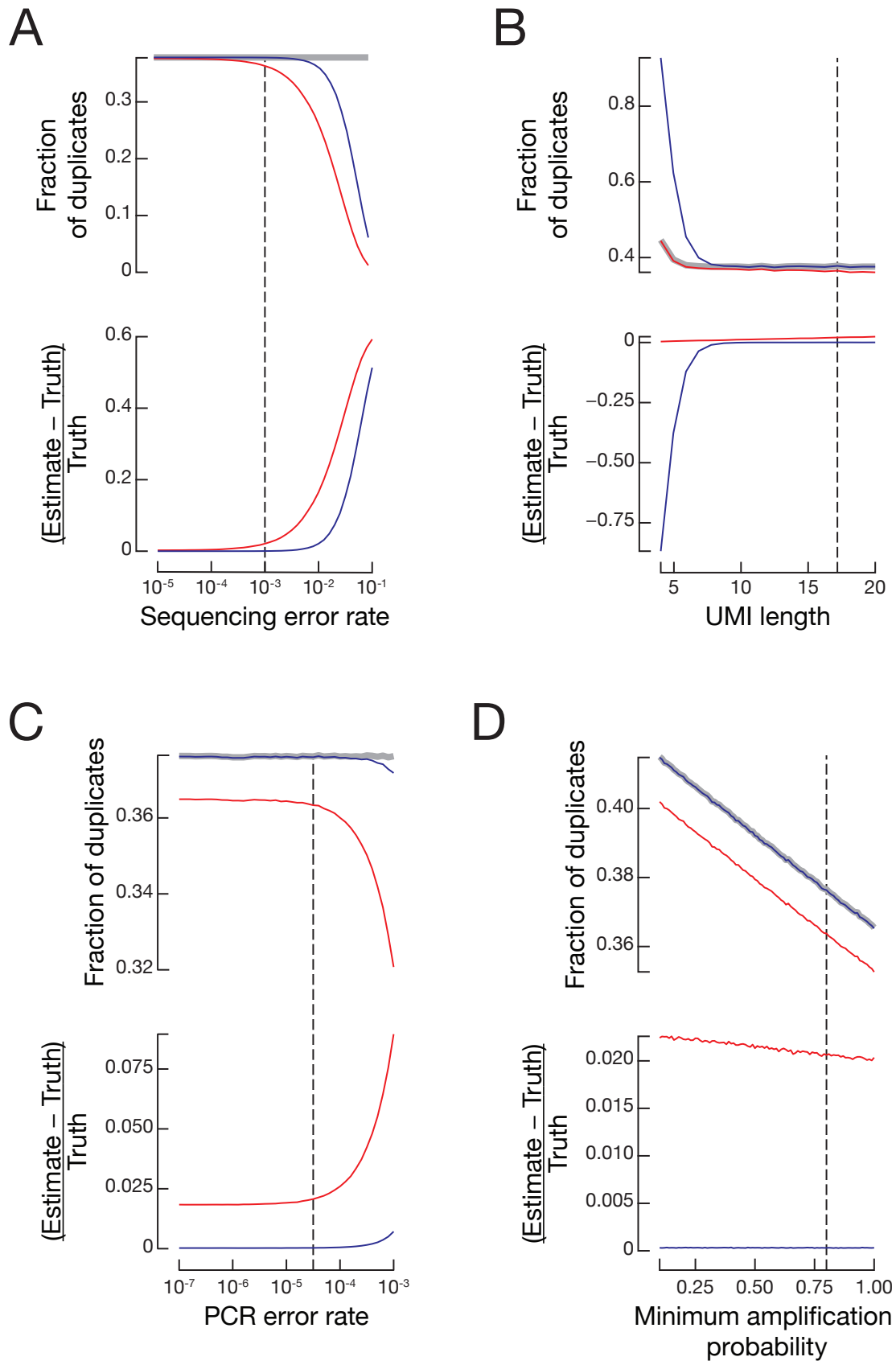

Supplement: Supplementary file 2 — Figure S1. Accuracy and fraction of duplicates for simulated data varying (A) sequencing error rate, (B) UMI length, (C) PCR error rate, or (D) minimum amplification probability. Each dotted line indicates the value for this parameter used in other simulations. (PDF 868 kb) [file 12864_2018_4933_MOESM2_ESM.pdf]
